# 884. Managing Appearance Concerns Early After Burn Injury: Ethical Considerations for Research and Clinical Practice

**DOI:** 10.1093/jbcr/irag033.370

**Published:** 2026-04-06

**Authors:** Laura Shepherd, Diana Harcourt, Fuschia Sirois, Andrew R Thompson

**Affiliations:** Nottingham University Hospitals NHS Trust, Nottingham, England; University of the West of England, Bristol, England; Durham University, Durham, England; Cardiff and Vale University Health Board, Cardiff University, Wales

## Abstract

Psychological distress associated with the visible changes to the body after a burn is common. This distress may be termed “appearance concern” which is estimated to occur in 1 in 3 adults after burns. Appearance concerns are known to start early post-burn (e.g., during hospitalization) and persist over time. Research suggests that appearance concerns are associated with significant psychological distress and functional impairment and can impact quality of life. Identification and management of appearance concerns at an early timepoint may significantly reduce the later psychosocial impact of injury. However, most research and practice in this area has been conducted following discharge from hospital and so there are gaps in the knowledge base and potentially missed clinical opportunities. Nevertheless, early psychosocial intervention and research poses ethical dilemmas, including: 1) Do the benefits of identifying and intervening with appearance concerns during hospitalization or early care outweigh the risks associated with doing so?; 2) Is research on appearance concerns (and interventions for this) early post-burn ethical and acceptable to patients and what can be done to optimize this?; and 3) What are psychological professionals’ views on the acceptability and feasibility of early psychological screening and interventions for appearance concerns being embedded into routine clinical practice, and what prevents this? Based on the evidence collected during studies in this area, this presentation will discuss the processes involved in carrying out this type of research to make the case that patients are likely to be willing to participate in research during hospitalization and early after discharge, and that patient advisory groups, patient and public involvement and co-development are key to conducting ethical and acceptable research. Finally, it will be argued that early screening for appearance concerns is on the balance of probabilities ethical practice, as is offering early psychological interventions (e.g., during hospitalization) as opposed to watchful waiting. Nevertheless, it is acknowledged that further research is needed.

